# A Novel Approach to Prognostic Factors and Risk Stratification in Pediatric AML: Case Report and Literature Review

**DOI:** 10.3390/ijms26199269

**Published:** 2025-09-23

**Authors:** Maria Leśniak, Anna Sekunda, Emilia Kamizela, Paulina Deleszkiewicz, Aleksandra Ozygała, Joanna Zawitkowska, Monika Lejman

**Affiliations:** 1 Student Scientific Society of Department of Pediatric Hematology, Oncology and Transplantology, Medical University of Lublin, 20-093 Lublin, Poland; marialesniak68@gmail.com (M.L.); annasekunda00@gmail.com (A.S.); kamizela2000@gmail.com (E.K.); 2 Department of Pediatric Hematology, Oncology and Transplantology, University Children’s Hospital in Lublin, 20-093 Lublin, Poland; paulina.deleszkiewicz@gmail.com; 3 Independent Laboratory of Genetic Diagnostics, Medical University of Lublin, 20-093 Lublin, Poland; aozygala1a@gmail.com (A.O.); monika.lejman@umlub.edu.pl (M.L.); 4 Department of Pediatric Hematology, Oncology and Transplantology, Medical University of Lublin, 20-093 Lublin, Poland

**Keywords:** AML, pediatric hematology, molecular genetics, cytogenetics, chemotherapy

## Abstract

Acute myeloid leukemia (AML), the second most common type of leukemia in children, is a heterogeneous disease known to be caused by genetic, epigenetic, and transcriptional changes, predominantly somatic and germline abnormalities. Despite significant improvement of overall survival rates, the prognosis of pediatric AML remains unfavorable in comparison with acute lymphoblastic leukemia (ALL), especially in relapsed or refractory settings. The current status and future directions are focused on establishing an accurate diagnosis and treatment strategies based on the genomic background. Next-generation sequencing (NGS) technologies enable a broader understanding of the basis of the disease for the purpose of determining pathology-associated mutations and additional prognostic biomarkers in pediatric AML. This review focuses on providing an overview of the known and possible prognostic factors, as well as genetic landscape of pediatric AML patients and how it can be used to accurately differentiate and risk stratify patients. It also presents potential candidate modifications for risk adjustment and targeted therapy. Furthermore, we describe in this article a case of a 22-month-old male patient with relapsed M5 high-risk group (HRG) AML with complex karyotype. Due to belonging to the HRG, as well as unsatisfactory chemotherapy response, the patient underwent matched unrelated donor (MUD) stem cell transplantation (SCT).

## 1. Introduction

Despite significant progress in identifying prognostic and predictive markers and the introduction of new therapies, mortality associated with AML remains relatively unchanged. AML continues to be one of the malignancies with the poorest prognosis, with a five-year overall survival (OS) rate of only about 33% [1,2,3]. Among pediatric patients, the overall survival rate has steadily been increasing and currently reaches approximately 75% in developed countries; by comparison, long-term survival rates were about 50% in the 1990s [4,5]. In children with ALL, this rate exceeds 90% [6].

AML is a heterogeneous, clonal malignancy originating from immature precursor cells of the myeloid lineage. It is characterized by uncontrolled proliferation and accumulation of blasts in the bone marrow, leading to impaired normal hematopoiesis [7,8]. Statistics from the National Institutes of Health (NIH) estimate that 22,010 new cases of AML will be diagnosed in 2025. AML is most commonly diagnosed among individuals aged 65–74, with this age group accounting for 26.6% of new cases. The incidence rate is 4.3 new cases per 100,000 men and women annually. Patients under the age of 20 represent only 4.1% of newly diagnosed cases [1,9]. The incidence of AML among infants, children aged 1 to 4 years, and in the 5–9-year-old age group is approximately 1.5, 0.9, and 0.4 cases per 100,000 individuals per year, respectively [10]. Among children, AML accounts for approximately 20% of all leukemia cases [11].

In children, there is no difference in the incidence of AML between sexes. While the development of AML can be associated with certain congenital chromosomal abnormalities (e.g., trisomy 21), inherited predisposition syndromes, germline mutations or structural gene alterations (such as those seen in inherited bone marrow failure syndromes), as well as acquired conditions or prior exposure to cytotoxic therapy, the vast majority of pediatric AML cases arise de novo, without a clearly identifiable cause. The pathogenesis is primarily driven by a stepwise accumulation of genetic and chromosomal alterations in immature myeloid precursor cells, ultimately leading to malignant transformation [12,13].

Despite effective initial treatment, approximately 24–50% of young patients experience a relapse, and the chances of long-term survival in this group are estimated to be only around 30% [5,14,15]. Clonal evolution plays a significant role in the relapse process, referring to dynamic changes in the genetic composition of the leukemic cell population in response to therapeutic pressure [16]. In patients who experience relapse, an emergence of new, previously undetectable subclones with mutations in genes such as *FLT3-ITD*, *NRAS*, *ASXL3*, or *KIT* is frequently observed [17,18]. Another factor contributing to AML relapse is the bone marrow microenvironment, which can protect leukemic cells from the effects of anticancer drugs. Studies indicate that the stromal and immunological components of this environment create a specific protective niche for leukemic blasts, significantly limiting the effectiveness of therapy [19]. In some patients, relapse may be caused by insufficient treatment intensity or the absence of hematopoietic stem cell transplantation (HSCT) in situations where it would have been indicated [20].

In current therapeutic protocols, pediatric patients with AML are divided into three main prognostic groups: favorable, intermediate, and adverse. Each of these categories is characterized by specific molecular and cytogenetic abnormalities that influence the course of the disease and the response to treatment [21].

## 2. Prognostic Factors

The overall prognosis of pediatric AML has improved significantly in recent years. One reason for this is an effective approach to risk stratification. In order to accurately assign patients to a specific risk group, it is necessary to start by identifying the various prognostic factors that will be associated, among other things, with the patient’s resistance to specific types of treatment. Such factors can be further divided into patient-related factors and disease-related factors.

### 2.1. Patient-Related Factors

In previous years, many studies reported the presence of a correlation between age and clinical characteristics of pediatric AML. Patients over the age of 10 had significantly worser outcomes and more treatment-related toxicities than their younger counterparts [22,23,24]. However, a study by Wang et al. supports the theory that infants, as well as young children, have a poorer overall prognosis. [25]. Infants and children aged 1 to 2 years oftentimes present adverse disease characteristics, such as higher white blood cell (WBC) counts, hepatosplenomegaly, or central nervous system (CNS) infiltration [26,27]. Furthermore, unfavorable outcomes of infant patients frequently stem from high-risk cytogenetic features associated with chemoresistance and heightened relapse rates. A retrospective study by Pawińska-Wąsikowska et al. analyzed AML patients aged 0 to 18 years treated in pediatric oncology centers in Poland from 2015 to 2022. The patients were risk-stratified based on cytogenetics of leukemic blasts and, among all four patient age groups, the infant group had the highest percentage of patients belonging to the HRG, 52% (*n* = 16) vs. 38.5% (*n* = 35) in the younger children group vs. 40.5% (*n* = 24) in the older children group vs. 33% (*n* = 13) in the adolescent group. Patients in the infant group obtained the lowest complete remission (CR) rate of 87%, compared to 95% in the younger children, however, the younger children group showed the highest relapse rate of 20.9%, compared to infants, older children, and adolescents (19.4% vs. 1.6% vs. 17.9%, respectively). More than half of the infant patients (51.6%) presented with the M5 AML subtype [28], which supports previous findings, indicating that infant AML is related to high frequencies of M4, M5, and M7 subtypes in the French–American–British (FAB) classification [26,27]. Unfortunately, the prognosis of such patients remains below expectations, with the OS rate at about 31% [29]. In a study by Liu et al. regarding patients with AML-M5, poorer OS and progression-free survival (PFS) were observed in high-risk patients with any of the following: *MLL-R*, *FLT3*-ITD, hyperleukocytosis, bone marrow (BM) blast percentage ≥ 70%, or age ≤ 3 years [30].

In light of recent reports, there is also a link between the race of patients and the course of their disease. Cytogenetic lesions in children with AML tend to differ by race-ethnicity. Regarding high-risk genetic features, Black patients were twice as likely to have −5/5q- or −7/7q- AML and five times more likely to have *KMT2A* rearrangement t(6;11)(q27;q23) AML in comparison with non-Black patients [31]. Moreover, as regards favorable genetic features, such as t(8;21) AML [31], *NPM1*-mutated *FLT3*-ITD^lo/no^ AML [32], and *RUNX1*::*RUNX1T1* fusion AML [33], the 3-year OS among Black patients still remained lower than that of non-Black ones. In addition to survival rates, time points of illness during which events contributing to poor survival occurred varied by race-ethnicity. Studies show that Black patients have the highest mortality from AML at diagnosis and in the first month after diagnosis, in contrast to non-Black patients, who have higher mortality rates later in the course of the disease [31,34,35]. A study by Lamba et al. suggests that racial disparities stem from dissimilarities in pharmacogenomics, as 51 of 70 examined Black patients presented with low scores of ACS10, cytarabine pharmacogenomics-based polygenic score, compared to 82 of 273 examined White patients. It was found that low ACS10 score correlated with considerably worse 5-year event-free survival (EFS) rates in comparison with those with high scores (42.4% vs. 70.0%) [36].

At present, it is emphasized in an accumulating number of studies that nutritional status of pediatric patients at the initial diagnosis of ALL impacts leukemia relapse and disease-related death [37,38]. Furthermore, previous studies in AML also showed that a body mass index (BMI) indicating either underweight or overweight at diagnosis correlated with poorer overall survival and higher treatment-related mortality, particularly due to infections and hemorrhage [39]. Although the negative impact of both underweight and overweight on the course of the disease has been confirmed, more recent studies do not clearly determine the impact of weight on specific long-term patient outcomes. One study found that, although a higher body weight was associated with lower disease resistance/relapse, it was also associated with a higher mortality rate during remission. Moreover, greater weight loss was significantly associated with death during the first remission [40]. In a study by Wen et al. children with low BMIs at the initial diagnosis and induction chemotherapy stages and with overweight at the initial diagnosis showed relatively lower 3-year EFS, 3-year relapse-free survival (RFS), and 3-year OS rates [41]. Despite a smaller AML patient cohort, the systematic review and meta-analysis summarizing results from 17 studies on the impact of body weight on pediatric patients with leukemia by Dong et al. revealed that underweight or obese patients showed a greater mortality risk and lower rates of EFS compared to children with normal BMIs. No difference in mortality risk was noted in overweight children. The risk of relapse was similar regardless of the BMI [42]. Fortunately, even though unhealthy BMI may well reduce the prognosis of pediatric AML patients, the recent progress in leukemia treatment and supportive care has significantly reduced the prognostic impact of the BMI at the time of the initial diagnosis [40,41].

AML typically presents in a sporadic context. However, in recent years there has been a growing understanding that certain inherited or de novo germline mutations significantly increase the incidence of myeloid neoplasms, such as AML [13,43]. In terms of genetic mutations, the development of AML has most often been associated with trisomy 21, as AML is 6–83 times more common among patients with Down syndrome than in the general population [44,45,46]. Moreover, there are some other genetic syndromes that have been linked with myeloid malignancy predisposition, such as Fanconi anemia [47,48], Bloom syndrome [49], Shwachman–Diamond syndrome [50], or Noonan syndrome [51], among others. A summary of selected, recently described germline mutations associated with the development of pediatric AML is presented in Table 1.

### 2.2. Disease-Related Factors

Hyperleukocytosis, typically defined as a WBC count greater than 100,000 cells/µL [88], occurring in 12.6 to 27.4% of pediatric AML patients [89,90], is a documented unfavorable prognostic factor [90,91], but its impact on the prognosis of the disease remains uncertain. Because of interaction between leukemic blasts and endothelial cells, such elevated WBC counts oftentimes lead to leukostasis, i.e., cells accumulating in the capillaries and blocking blood flow, disseminated intravascular coagulation (DIC), secondary hemorrhages, respiratory and neurological complications, and/or tumor-lysis syndrome (TLS), in comparison to patients with lower WBC counts [89,92]. In a study by Zhang et al. analyzing pediatric AML patients with different criteria of hyperleukocytosis, patients with WBC counts of 50,000–100,000 cells/µL and WBC counts ≥100,000 cells/µL had similar CR rates (71.4% vs. 75.4%) and 5-year EFS rates (78.0% ± 5.3% vs. 81.1% ± 2.9%). Patients with hyperleukocytosis presented a higher rate of relapsed/refractory disease and mortality than those without hyperleukocytosis [90]. Another study found an association between hyperleukocytosis and significantly inferior 5-year EFS and a trend of inferior OS of patients compared to those without hyperleukocytosis [93]. Moreover, significant leukocytosis was found to be associated with an age below 1 year and corresponded to a higher proportion of FAB subtypes of M1, M4, M5, intermediate chromosomes, *FLT3*-ITD/TKD, *CEBPA* mutation, inv (16), MLL, and gene fusion of *CBFB-MYH11* and *NUP98-NSD1* [90,93,94]. It is important to note that lower EFS rates caused by greatly elevated WBC counts were found irrelevant of cytogenetics status [93] but were connected with the age of patients at the time of diagnosis, with children aged 1 to 9 years having higher 5-year EFS rates [91]. Additionally, a WBC count of ≥100,000 cells/µL at the time of diagnosis was a risk factor of disease relapse [95].

### 2.3. Genetics of Pediatric AML

Despite remarkable AML diversity, encompassing everything from clinical manifestations to morphology, some chromosomal and molecular alterations are recurrent and characteristic of pediatric AML. Such alterations include t(15;17)(q24.1;q21.2) with a *PML-RARA* fusion gene [96], AML with core-binding factor (CBF) with *RUNX1-RUNX1T1* and *CBFB-MYH11* fusion genes [97], 11.q23 rearrangements (*KMT2A*) [98], inv(16) (p13.3q24.3) with fusion gene *CBFA2T3-GLIS2* [99], 11p.15 rearrangements (*NUP98*) [100], aneuploidy in the form of monosomy 5/5q-, monosomy 7, or abnormal 12p [101].

**Figure 1:**
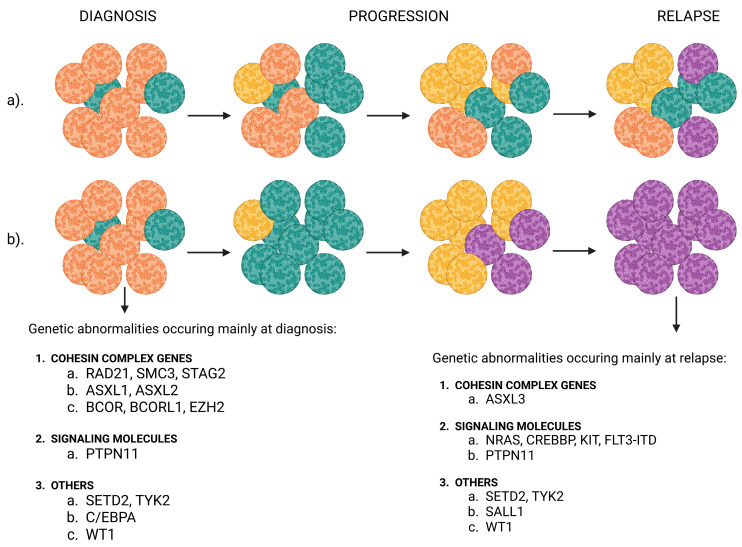
Models of clonal evolution in AML and mutations in specific gene families typical during clonal evolution. (**a**) Linear evolution describes the sequential acquisition of mutations. The relapse-originating cell is part of the major clone at diagnosis. (**b**) Branching evolution describes the eradication of the major clone and subsequent outgrowth of a secondary clone. The relapse-originating cell is part of a subclone at diagnosis. Created in https://BioRender.com, accessed 16 July 2025.

On the other hand, the heterogeneity of AML is justified by the concept of clonal evolution of leukemic blasts [27,98,102], in which, during the course of the disease, subclones derived from a common ancestral clone are selected and expanded, acquiring distinct mutations that confer a survival advantage, leading to genetic differentiation within the cell lineage. Alongside disease progression, individual AML blast populations may undergo distinct patterns of clonal evolution, linear and branching, as well as an abundance of mutations at various time points [103,104]. Several studies [105] have analyzed whole-exome sequencing (WES) samples of blasts from pediatric AML patients at diagnosis, as well as later in the disease course, during remission and relapse. It has been shown that most dominant variants that arise during leukemia development persist from diagnosis until relapse, but subclonal modifications can also occur and develop later. This suggests that the genomic landscape at the time of relapse differs from the initial clone and can be influenced by the treatment [105,106]. Even though it is difficult to find recurrent single mutations, there are recurrent mutations in specific gene families [105]. Different concepts of clonal evolution of blasts and typical mutations [105,106,107,108] are presented in Figure 1.

### 2.4. Measurable Residual Disease

The term measurable residual disease (MRD) refers to the presence of low, otherwise undetectable, levels of blast cells, from below 1:10^4^ to 1:10^6^ WBCs, and is an independent prognostic factor assessed after the diagnosis of AML, crucial for patient evaluation, risk stratification, and treatment planning [109]. After MRD evaluation, patients are typically divided into two groups: MRD < 0.1% and MRD ≥ 0.1%, though some studies used three categories when describing their MRD risk groups: MRD < 0.1%, MRD 0.1–1%, and MRD ≥ 1% [110,111]. An attainment of MRD negativity indicates the patient’s early treatment response [109] and correlates with greater long-term survival [110,112]. In a systematic review and meta-analysis of 81 publications regarding AML patients, the estimated 5-year disease-free survival (DFS) and OS rates in the MRD-negative patient group were significantly higher than in that with positive MRD, i.e., 64% vs. 25% and 68% vs. 34%, respectively [112]. Similarly, a systematic review of 13 publications solely regarding pediatric AML patients supports the statement that MRD-negative patients consequently obtain better clinical outcomes [110] It should be noted that the absence of MRD at assessment does not exclude a risk of disease relapse, as the relapse rate in the pediatric population who obtain MRD negativity after the induction cycle ranges between 20% and 25% [113]. MRD can be assessed using various tools, including the most commonly used multiparameter flow cytometry (MPFC). New methods and protocols are emerging, allowing MPFC to be supplemented with molecular techniques such as sensitive quantitative or digital droplet polymerase chain reaction (qPCR, ddPCR) methods and, more recently, error-correcting NGS technologies to identify gene mutations associated with AML. Unfortunately, these methods have not yet been standardized qualitatively or quantitatively, which hinders their application in clinical practice [114]. Major MRD-assessment technologies are showcased in Table 2.

## 3. Risk Stratification in Pediatric AML

Risk stratification is one of the key components of effective AML treatment. Current risk stratification systems in pediatric AML are based on a combination of leukemia-specific cytogenetic and molecular abnormalities, along with treatment response assessed through MRD evaluation. In 2017, an updated version of the European Leukemia Net classification (ELN 2017) was published, introducing a standardized risk categorization for AML, dividing patients into three main prognostic groups: favorable, intermediate, and adverse [21]. Each group is defined by a distinct genetic profile that plays a crucial role in disease progression, anticipated treatment response, and the selection of therapy intensity [21].

One of the best-characterized chromosomal abnormalities in AML is the t(8;21)(q22;q22) translocation, typically associated with the M2 morphological subtype. This translocation results in the formation of the *RUNX1*::*RUNX1T1* fusion gene, which was among the first to be utilized in clinical practice for the detection of MRD [122]. The t(8;21) translocation occurs in approximately 10–12% of pediatric patients with AML [97]. This aberration is typically associated with a reduced white blood cell count and the presence of large blast cells characterized by basophilic cytoplasm, numerous azurophilic granules, and Auer rods [123,124]. Treatment of AML patients with the t(8;21) mutation typically involves induction regimens based on anthracyclines and cytarabine, followed by two to four cycles of consolidation therapy using high-dose cytarabine. In selected cases, treatment may be supplemented with gemtuzumab ozogamicin (GO) [125].

The chromosomal abnormality inv(16)(p13;q22) or t(16;16)(p13.1;q22) leads to the formation of the *CBFβ*-*MYH11* fusion gene [126]. The *MYH11* gene encodes the smooth muscle myosin heavy chain (SMMHC), whereas the *CBFβ* gene encodes the β subunit of the CBF [127]. In patients with the inv(16)(p13;q22) translocation, markedly elevated white blood cell and monocyte counts are often observed, along with the presence of eosinophils displaying abnormal morphology. Eosinophils at various stages of maturation can be seen. These features are characteristic of the leukemia subtype known as AML M4Eo [128].

AML with t(8;21)(q22;q22) and AML with inv(16)(p13.1q22)/t(16;16)(p13.1;q22) are collectively referred to as CBF-AML [129]. CBF-AML accounts for approximately 15% of AML cases in adults and 25–30% in children [130]. The CBF AML subtype is associated with a better prognosis, but it exhibits similar relapse rates of approximately 30%, comparable to other AML subtypes [95,131].

Other abnormalities classified as favorable prognostic factors include mutations in the *CEBPA* gene and the *NPM1* gene. Both of these mutations are considered cytogenetically normal aberrations in AML. CEBPA is a transcription factor that plays a key role in cytodifferentiation, that is, maturation of myeloid cell precursors during hematopoiesis. It regulates the expression of genes specific to granulocytes, enabling their proper differentiation [132]. *CEBPA* mutations occur in 5–10% of pediatric patients, with double mutants showing an 80% 5-year OS rate [133,134]. Nucleophosmin 1 (NPM1) is a protein responsible for transport between the nucleus and the cytoplasm. It is primarily localized in the nucleolus, where it performs a variety of essential functions [135]. Mutations in the *NPM1* gene occur in various variants, but all involve an insertion in its C-terminal region. This alteration causes abnormal translocation of the protein from the nucleus to the cytoplasm, a phenomenon referred to as the NPMc+ phenotype [136].

FLT3 is a receptor tyrosine kinase activated by a transmembrane ligand, physiologically expressed primarily on hematopoietic stem and progenitor cells. It plays a critical role in regulating the early stages of myeloid and lymphoid lineage development. Mutations in *FLT3* lead to constitutive activation of its tyrosine kinase activity, resulting in uncontrolled cellular proliferation. The two major types of *FLT3* mutations are internal tandem duplications (*FLT3*-ITD) within the juxtamembrane domain and point mutations or deletions in the tyrosine kinase domain (*FLT3*-TKD) [137]. *FLT3*-ITD mutations are common in pediatric AML, accounting for approximately 10–20% of pediatric AML cases. The 5-year OS is only 30–40% [134].

The *KMT2A* gene (formerly *MLL*), located on chromosome 11q23, encodes a protein involved in the regulation of developmental gene expression, including key genes of the *HOX* family, which are critical for hematopoietic cell differentiation [138,139]. Translocations involving the 11q23 region are associated with an aggressive disease course, marked leukocytosis, and an increased risk of relapse, making this molecular variant a significant prognostic factor [138]. One such rearrangement is the t(11;19)(q23;p13.3) translocation, resulting in the *KMT2A*::*MLLT1* fusion, which is found in 1% of infants and 6% of children and adolescents with *KMT2A*-rearranged AML [140]. Fusion with the *MLLT1* (*ENL*) gene results in the persistent recruitment of active transcriptional complexes through the YEATS domain, which recognizes acetylated lysine residues on histones. This leads to constitutive activation of gene expression programs that promote oncogenic transformation [141]. Currently, *KMT2A* fusions, including those involving *MLLT1*, are the focus of intensive research into targeted therapies. Of particular interest are menin inhibitors, which disrupt the interaction between MLL1 and the menin protein, leading to the suppression of aberrant *HOX* gene expression, as well as DOT1L inhibitors, which target the H3K79 methyltransferase enzyme recruited by *KMT2A* fusion complexes [139,142,143].

A summary of the ELN 2017 classification for risk stratification, including factors not listed above, is presented in Table 3.

## 4. Risk-Adapted Treatment Approach

### 4.1. Upfront Therapy

Although upfront therapy in pediatric AML is a widely discussed topic among researchers, its backbone remains the same. The common standard comprises a chemotherapy regimen based on a combination of anthracycline with cytarabine [148]. Chemotherapy is quintessentially divided into two main stages, initiation, which aims at achieving initial remission, followed by consolidation, alternatively named intensification, which consists of additional courses of chemotherapy to eradicate residual disease. During the consolidation phase, high-risk patients receive allogenic HSCT (allo-HSCT) if a desirable donor is found and the patient achieved MRD negative remission [2,134]. Predominantly, the induction phase is based on the “7 + 3” regimen, which consists of a low-to-intermediate dose of cytarabine for seven days, followed by anthracycline for three days. This combination can be supplemented by a third medication, mostly etoposide [12]. However, the exact role of etoposide in the regimen is not yet fully understood and, what is more, in some studies it was decided not to include etoposide in the induction regimen [149]. The consolidation stage requires a nearly identical set of agents, i.e., mostly high-dose cytarabine with an optional addition of anthracyclines or other drugs. The exact number of chemotherapy courses, as well as inclusion of specific supplementary agents, is yet to be determined [12]. Anthracyclines used in this context include daunorubicin, idarubicin, and mitoxantron [150]. A newer approach considering anthracyclines promotes using liposomal daunorubicin, which was developed with an aim to allow dosage increase without intensifying adverse effects, mainly cardiotoxicity [151]. Studies have found that, compared to daunorubicin, including idarubicin and mitoxantrone in chemotherapy regimens did not provide improved treatment results, yet liposomal daunorubicin was associated with lower treatment-related mortality (TRM) in comparison to idarubicin [149]. Apart from anthracyclines, cytarabine is also available in a liposomal formulation. A combination of both agents, called CPX-351 together, with a daunorubicin to cytarabine molar ratio of 1:5, ensures elongated exposure time. The effectiveness and safety of such a solution was proven in adult patients with AML and current studies focusing on children also seem to be obtaining promising results after including CPX-351 in upfront therapy [152,153]. Another emerging approach to upfront therapy involves incorporation of GO, toxin-conjugated humanized IgG4 anti-CD33 monoclonal antibody, into conventional chemotherapy [154]. CD33 expression is highly associated with AML as it appears in up to 80% of patients but also indicates unfavorable prognosis. However, based on conducted research, GO improves EFS of pediatric patients with freshly diagnosed AML when added to induction and second courses of the consolidation phase of chemotherapy. This has led to approval by the Food and Drug Administration (FDA) for GO to be used in upfront treatment of AML in patients over 1 month old and for relapsed or refractory AML in patients over 2 years old [12,155,156]. Children’s Oncology Group (COG) suggests implementing GO in all AML patients, regardless of CD33 expression level [157]. Another promising approach targeting better treatment results in children with high allelic ratio (HAR) suffering from FLT3/ITD+ AML is an addition of sorafenib, multikinase type II inhibitor, to conventional chemotherapy. Such a combination provided a significant advancement in both EFS and DFS rates, with additional decline in relapse risk. Incorporations of GO or kinase inhibitors, such as sorafenib, into standard regimens are recommended to become a standard of care, however further research is still needed [158,159].

Nevertheless, not all novel strategies yield favorable outcomes. In trials in implementing bortezonib in conventional chemotherapy, not only did it not improve remission reduction rate but it also boosted treatment-related toxicities [160]. Another disappointing trial regarded inclusion of clofarabine, a second-generation purine nucleoside analogue, in the induction regimen (CLOF-based regimen) as it did not provide any encouraging conclusions [134,161].

Several studies showed a great advantage from intensification of conventional chemotherapy, especially since children tolerate high-intensity regimens better than adults. However, TRM was reported to be between 6% and 10% and short-term and long-term toxicity was found in around 40% of pediatric patients, which indicates that the intensity of conventional chemotherapy has reached its ceiling and the development of new approaches is mandatory [148,157]. It is also alarming that up to 40% of pediatric patients with de novo AML face relapse, despite intensified chemotherapy and expanded use of upfront allo-HSCT. The treatment of such relapses still poses a burdensome challenge [18]. Taking these facts into account, alternative treatment methods are being developed, with strong emphasis on personalized molecular targeted therapy, as well as immunotherapy [162].

### 4.2. Hematopoietic Stem Cell Transplant

Although there is ongoing research focused on finding newer treatment possibilities, allo-HSCT remains a necessary option for a certain group of patients [163]. The exact indications are not strictly set but most studies suggest that children with high-risk AML or relapsed AML are the most suitable recipients of allo-HSCT. Yet, a consensus is still missing for intermediate-risk AML in the context of allo-HSCT usage [157]. Since roughly 25% of pediatric AML is associated with disease relapse or is classified as high risk, allo-HSCT is an option for a prominent group of patients [164]. Allo-HSCT is generally performed in the first complete remission (CR1) as treatment augmentation when patients present unfavorable prognostic factors, including certain cytogenetic lesions, or their response to the induction phase of therapy is not satisfactory [165]. Research indicates that, with an increasing number of children undergoing HSCT, survival rates for AML are improving. This phenomenon is possible due to improving supportive care and better accessibility of donors [166]. The general objective of HSCT is to lessen relapse prevalence, improve graft-versus-leukemia (GVL) results and decrease graft-versus-host disease (GVHD), as relapse after HSCT is still a great issue leading to treatment failure, with more research regarding pre- and post-HSCT interventions being needed [167]. The cytotoxic effect of conditioning regimens and GVL’s effect from cytotoxic immune cells derived from the donor constitute the base for therapeutic outcomes of HSCT. If the transplant is performed during the first or second CR, DFS is expected to be at 60–70%, whereas TRM would be at 10–15% [168]. Presently, the standard for conditioning before HSCT includes either total body irradiation (TBI) or chemotherapeutics consisting of mixed alkylating agents. The most significant challenge is to find an accurate balance between toxic and therapeutic effects, as not conditioning forcefully enough can lead to disease relapse or graft rejection, whereas too aggressive conditioning can be the reason for tissue disruption or both acute and late life-threatening complications [169]. Standard conditioning with chemotherapeutics comprises regimens such as busulfan–cyclophosphamide (BuCy), busulfan–cyclophosphamide–melphalan (BuCyMel) and clofarabine–fludarabine–busulfan (CloFluBu). Results from studies comparing those regimens suggest that BuCy is associated with lower leukemia-free survival (LFS) as the relapse rate was higher, with younger age serving as a predictor of relapse, whereas ByCyMel and CloFluBu provided corresponding LFS rates. Moreover, CloFluBu yielded a decreased rate of acute GVHD, which correlated with busulfan therapeutic drug monitoring (TDM). As a result, CloFluBu is considered a promising, TBI-free regimen that is characterized by acceptable toxicity and a strong antileukemic effect [169,170]. In most cases the choice between myeloablative conditioning (MAC) and reduced-intensity conditioning (RIC) depends on the child’s general condition, comorbidities, relapse risk, and the ability to tolerate toxicity. If the patient does not show a genetic predisposition for increased toxicity, MAC is usually chosen for the first HSCT and RIC is chosen for the second HSCT or for patients with coexisting conditions [167]. With MAC, TRM is at a high rate, especially late TRM, while RIC is generally related to lower TRM, however, because of poorer disease management, the relapse risk is much more significant. Research suggests that, taking overall survival into consideration, results for MAC were considerably more favorable. After a long-term follow-up the children treated with the MAC regimen exhibited better survival rates than those receiving RIC, including children who were suitable for both MAC and RIC [171]. Despite advancement in conditioning regimens, relapse risk is still unsatisfactorily high and new approaches are being proposed. One approach indicates using prophylactic/pre-emptive low-dose azacitidine and donor lymphocyte infusion (DLI) as part of a maintenance regimen after HSCT to boost the GVL effect and consequently reduce relapse occurrence. The study proved that the method was not only safe but patients receiving maintenance also had improved overall survival as well as PFS compared to patients without this regimen [172]. Another novel strategy concerns an addition of targeted therapeutics, such as venetoclax and daratumumab, to the conditioning regimen, basing on the expression level of B-cell leukemia/lymphoma-2 (BCL-2) and CD38, as these targets frequently occur in pediatric AML. Although this preparative regimen was proved safe, there was no proof of long-term enhancement of post-transplant results [173].

### 4.3. Molecular Targeted Therapy

Research into targeted therapy aims at finding more personalized and accurate treatment strategies that work alongside conventional chemotherapy, intensifying its effects [151]. *FLT3* mutations are among the most common genetic lesions in pediatric AML and are found in nearly 20–25% of patients [150]. The *FLT3* gene encodes a transmembrane ligand-activated receptor tyrosine kinase, which is expressed by hematopoietic stem and progenitor cells in regular conditions, as it regulates hematopoiesis through several signaling pathways, such as PI3K, RAS, and STAT5. The two types of these mutations are an internal tandem duplication (*FLT3*-ITD) within the juxtamembrane (JM) domain and a mutation of the tyrosine kinase domain (*FLT3*-TKD). An interaction between FLT3 ligand and FLT3 receptor causes pathway activation leading to transcriptional changes which in the case of *FLT3*-ITD and *FLT3*-TKD mutations cause constant activation of FLT3 kinase and proliferation and survival of AML cells [174,175].

BCL-2 refers to a family of proteins involved in innate, mitochondrial apoptotic pathway regulation, whereas Myeloid cell leukemia 1 (MCL-1) is a member of that family. Both are antiapoptotic (prosurvival) proteins frequently present in hematologic malignancies, as their overexpression causes disruption of apoptosis by BCL-2 seizing proapoptotic BCL-2 homology 3 (BH3)-only proteins (BIM, BAD, BID), which suppresses oligomerization of pore-forming proteins (BAX and BAK) and mitochondrial outer membrane permeabilization (MOMP), thus promoting tumorigenesis and impaired reaction to chemotherapy. BCL-2 inhibitors aim at preventing this process [176,177].

**Figure 2:**
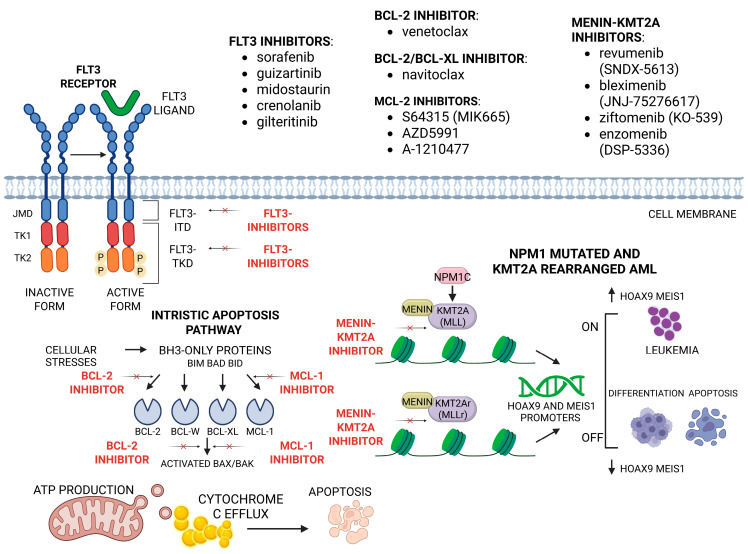
Mechanisms of action of FLT3 inhibitors, BCL2 inhibitors, and menin-KMT2A inhibitors. Created at https://BioRender.com, accessed 16 July 2025.

Menin inhibitors are another possible targeted treatment strategy, designed primarily for patients with *KMT2A* rearrangements (*KMT2Ar*) or *NPM1* mutations. Menin is a protein found in the cell nucleus, encoded by the *MEN1* gene that facilitates the assembly and stabilization of various transcriptional and chromatin-modifying complexes. Notably, it interacts with the KMT2A (MLL) fusion protein complex, a critical epigenetic regulator involved in transcriptional control, particularly in leukemogenesis, using transcription factors *HOXA9* and *MEIS1.* Inhibition of this interaction inhibits AML cell proliferation, thus reducing leukemia progress [151,178]. Mechanisms of action of FLT3 inhibitors, BCL2 inhibitors, and menin-KMT2A inhibitors and novel drugs [151,177,178,179,180,181] are shown in Figure 2.

**Figure 3:**
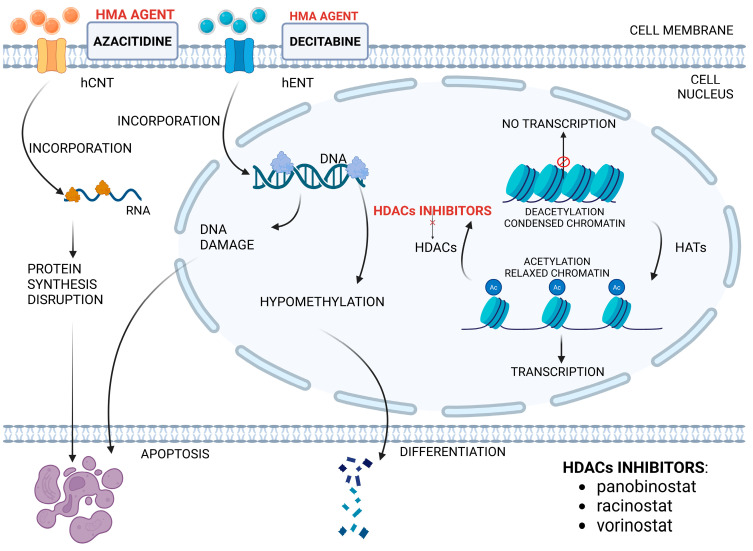
Mechanisms of action of epigenetic modifiers. Created at https://BioRender.com, accessed 16 July 2025.

Since mutations of epigenetic regulators, as well as epigenetic alterations, are part of pediatric AML pathogenesis, epigenetic therapeutics are being developed, including hypomethylating agents (HMAs) and histone deacetylase (HDAC) inhibitors (HDACis). HMAs azacitidine (AZA) and decitabine (DEC) exert their antileukemic effects by reinstituting epigenetically silenced tumor suppressor genes, reversing gained DNA methylation patterns characteristic of malignant cells, and modulating the activity of mutated epigenetic enzymes. HDACis present a distinct mechanism of action, focusing on histone (de)acetylation modulation. Histone acetylation is part of several processes, including replication, DNA repair, apoptosis, or chromatin packing and cell differentiation. It is stabilized by histone deacetylases (HDACs) and histone acetyltransferases (HATs), adjusting gene expression. By suppressing HDACs, HDACis contribute to maintaining open chromatin conformation [182,183]. Mechanisms of action of epigenetic modifiers and novel drugs [150] are shown in Figure 3.

### 4.4. Immunotherapy

AML shows susceptibility to immune-based therapies, due to its distinct immunological profile, making it a promising target for immunotherapy. AML cells express both major histocompatibility complex (MHC) class I and II molecules, which is related to being recognized by T cells and vulnerable to T-cell-mediated cytotoxicity. The inherent immunological cooperation between myeloid cells and the immune system further supports the development of immunotherapeutic strategies aimed at targeting leukemic blasts. The effectiveness of conventional AML treatments, such as allo-HSCT and DLI, strengthens the role of immune effector cells, particularly T cells and natural killer (NK) cells, in detecting and eliminating leukemic cells, therefore highlighting AML’s sensitivity to immunotherapy [184].

**Figure 4:**
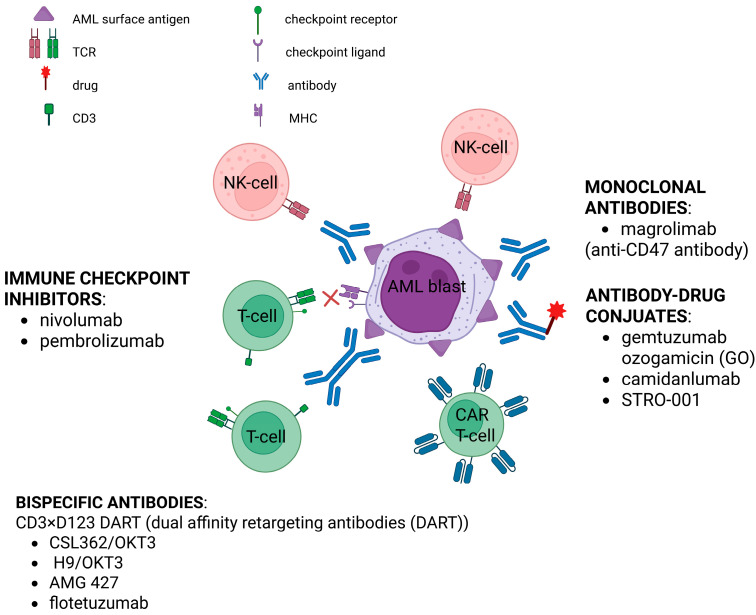
Immunotherapeutic approaches to AML. Created at https://BioRender.com, accessed 16 July 2025.

There are currently several immune-based strategies being developed, including monoclonal and bispecific antibodies, chimeric antigen receptor T-cell (CAR-T) therapy, antibody–drug conjugates (ADCs), and application of checkpoint inhibitors. For antigen-specific monoclonal antibody therapy, CD33 and CD123 are the most common targets, as they are frequently expressed in AML and, therefore, have a significant potential to bring satisfactory treatment results. Research also includes using CD47 as a target, as leukemia cells tend to avoid phagocytosis, due to overexpression of “don’t eat me signals”, such as CD47. A further evolution of antibody-based regimens focuses on monoclonal antibodies developed with an aim to enhance antitumor activity by T-cell engagement. Such antibodies are called bispecific because as they present dual affinities for tumor cell antigens and immune effector cell antigens and comprise the minimal binding domains of two different antibodies on one, such as bispecific T-cell-engager (BiTE) antibodies, or two, such as dual-affinity retargeting (DART) antibody polypeptide chains. An application of ADCs is another method based on monoclonal antibodies, as they are connected to a cytotoxic payload with a cleavable linker. Such arrangement lets particularly cytotoxic drugs enter leukemia cells directly, resulting in cell death without unnecessary off-tumor toxicity. A newer approach involves using checkpoint inhibitors in patients with pediatric AML, because of their ability to boost immune reaction to tumor cells. Although this method has been proven successful in adult solid malignancies, it still needs to be further examined in AML. At present, nivolumab and pembrolizumab, anti-PD-1 antibodies, as well as ipilimumab, targeting CTLA-4, are being tested in clinical trials. CAR-T therapy is currently being studied as a potential immunotherapy strategy, however, the lack of a unique cancer surface marker poses a challenge. As previously mentioned, CD33 and CD123 are among the most common markers and constitute a potential target for such therapy. There are particularly high hopes for C33 as it is not present on hematopoietic stem cells and GO showed satisfactory outcomes in AML which, taken together, gives a base for more advanced investigations. Lastly, NK cells also represent a promising immunotherapy target, serving several crucial functions in the immune system, enabling the use of universal donors and being related to a lower risk of inducing GVHD. Ongoing clinical trials are aimed at clarifying the design of CAR-T and CAR-NK cells, incorporating multispecific targeting approaches, mitigating treatment-related toxicities, and enhancing strategies to prevent disease relapse [151,156,165,184,185]. Immunotherapeutic approaches and novel drugs [150,165,186,187,188,189] are shown in Figure 4.

## 5. Future Directions

Although various genetic lesions are already part of risk stratification in children with AML, many mutations are yet to be revealed. A new study, using targeted NGS, found a novel recurring mutation in the *BCORL1* gene, which was present in 9% of patients at diagnosis. The same study also identified *CUX1*, *KDM6A*, *PHF6*, and *STAG2* mutations, whose frequencies were significantly higher than those in similar earlier reports. Such information is crucial for understanding the heterogenous genetic foundation of childhood AML, leading to better molecular characterization and more efficient risk ranking [190].

Hypodiploidy is a risk factor that has not yet been independently analyzed in pediatric AML. It is defined as modal numbers (MNs) of 45 and less and found in nearly 2% of cases. MNs of 43 and 44 are related to unsatisfactory survival outcomes, particularly monosomal karyotype or monosomy 9, whereas an MN below 43 has not been discovered. Hypodiploid AML is described as a rare subgroup that showed unfavorable results, even after HSCT in CR1. Consequently, children from this group should be more precisely assigned to risk groups, due to hypodiploidy being a high-risk factor, and alternative therapies should be explored since those patients might not benefit from conventional strategies [191].

One of the novel diagnostic approaches includes using targeted NGS of circulating tumor DNA (ctDNA), held in circulating free cell DNA (cfDNA) that is released as a result of necrosis or apoptosis of a tumor cell, as a sample for mutation recognition. Studies show that ctDNA not only provides a mirrored image of genomic patterns from bone marrow but there is also a subset of mutations that is discovered only through ctDNA detection. This method is relatively non-invasive and can be complementary to bone marrow biopsy for gathering valid information that can further refine treatment strategies for pediatric AML, however, extended research is still needed [192].

There is an additional marker that has both diagnostic and prognostic capacity, that is, the expression of microRNA-29a (miRNA-29a) and microRNA-100 (miRNA-100). miRNA is an epigenetic controller of regular cell processes, and its malfunction plays a major role in the progression of several pathologies, including hematological malignancies. Two distinct patterns of expression were observed for miRNA-29a and miRNA-100 in a 2022 study by Said et al. Children with AML presented explicitly increased circulation of miRNA-100, when compared to a healthy population, whereas circulation of miRNA-29a was noticeably lower in AML patients. Both markers are said to be predictors of unsatisfactory results after standard therapy, which can be of help when selecting the most suitable treatment strategy [193].

A newly discovered mechanism called cuproptosis impacts AML biology and has a potential to become a prognostic predictor and target for therapy. It is a type of programmed cell death that is prompted by accumulation of intracellular copper ions. Cuproptosis-related genes (CRGs), such as *CNN3* and *LGR4*, are associated with modulation of AML progression, with *CNN3* being correlated to unfavorable prognosis, which can be a prospective therapeutic point of interest, along with advancing risk assessment [194].

In the search for a new treatment strategy, in pre-clinical and clinical results, CD74 expression occurred as an encouraging target. CD74, an invariant chain of MHC-II, is associated with promoting antigen presentation, in addition to being part of stem cell maintenance, activation of macrophages and monocytes, as well as T-cell and B-cell function. It has been observed that CD74 expression was significantly higher in several hematopoietic malignancies, including pediatric AML, compared to normal myeloid progenitor cells, making it a potential therapeutic target. Another suggested application is using CD74 in monitoring MRD after therapy. It has also been found that increased CD74 expression is correlated to positive prognostic genetic lesions, and such patients tend to achieve higher EFS, yet still 50% of those patients face adverse events. Despite very promising findings, further data is required to develop more specific utilization of such knowledge [189].

One of the biggest challenges is the fact that pediatric AML is a widely immunophenotypically and biologically heterogenous disease and there are significant differences when compared to adult AML. Thus, more children-focused studies are needed to allow even more precise and adequate risk stratification, along with the choice of optimal treatment strategies [19,195].

## 6. Case Report

### 6.1. Medical History and Complaints at Presentation

A 15-month-old male patient with no previous medical history was admitted to the University Children’s Hospital in Lublin with complaints of anuria, accompanied by increased thirst and weakness that started the previous day. According to the parents, the boy experienced profuse sweating, which had been present for about two months.

### 6.2. Results at Admission

On admission, the patient was in semi-good clinical condition, visibly apathic, with skin pallor and minor petechiae present on lower limbs and lower border of the liver palpable over 3 cm below the costal margin. Initial laboratory tests revealed severe hyperleukocytosis of 166,380 cells/μL, automated peripheral blood count showed 30% neutrophiles, 15.4% lymphocytes, and 53.8% monocytes, plus mild thrombocytopenia with platelet count 92,000 cells/μL. Abdominal ultrasonography was unremarkable. The patient was admitted to the Department of Pediatric Hematology, Oncology and Transplantology with suspected hematological malignancy.

**Figure 5:**
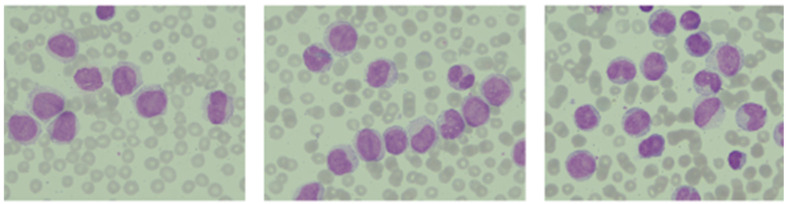
Peripheral blood smear findings of the AML case: myeloblasts in the cytoplasm (Wright-Giemsa stain).

A peripheral blood smear showed about 88% myeloblasts (Figure 5). Because of the elevated WBC count, peripheral blood flow cytometry was performed instead of bone marrow sampling. The cytometric phenotyping showed precursor cells with the following phenotype: CD33 98%, CD117 30%, CD45 88%, CD64 97%, which confirmed the diagnosis of AML M4/M5 according to the former FAB classification.

### 6.3. Patient Management During Hospitalization

The patient was treated with pre-phase chemotherapy consisting of cytarabine (1 × 20 mg/m^2^), followed by ICE (idarubicin 10 mg/m^2^/day, days 1–3 + cytarabine 200 mg/m^2^/day in continuous infusion, days 1–7 + etoposide 100 mg/m^2^/day, days 1–5) for induction treatment.

Hematological remission was achieved after the first induction cycle, twenty-eight days afterwards. BM aspirate revealed 4.2% myeloblasts and 0.003% flow-cytometry-measurable residual disease (FC-MRD). A molecular analysis confirmed the presence of a *KMT2A::MLLT1* fusion gene and, on this basis, the patient was then later classified into the AML with *KMT2A*-rearrangement diagnostic category, according to the 2022 World Health Organization (WHO) Classification of Pediatric Tumors. Cytogenetic analysis using GTG banding revealed a complex karyotype. Metaphase cells from short-term 24 h unstimulated cultures were examined. Bone marrow cytogenetic testing exhibited a complex karyotype described according to the International System of Human Cytogenomic Nomenclature (ISCN) 2020 as 47,XY,del(1)(p32),+8,der(11)t(11;14)(q13;q32),der(14)t(14;11)(q32;q13)t(11;19)(q23;p13),der(19)t(11;19)(q23;p13)[25]. The boy was classified according to the high-risk (HR) Associazione Italiana Ematologia Oncologia Pediatrica-Berlin, Frankfurt, Münster-AML-2020 (AIEOP-BMF-AML-2020) protocol, and treatment was administered according to the AIEOP-BMF-AML-2020 clinical trial group A.

The patient proceeded to receive a second induction chemotherapy course with HAM (cytarabine 3000 mg/m^2^ every 12 h, days 1–3 + mitoxantrone 10 mg/m^2^/day, days 3, 4), followed by HAE (cytarabine 3000 mg/m^2^ every 12 h, days 1–3 + etoposide 125 mg/m^2^/day, days 2–5) chemotherapy. A second BM aspirate revealed 4.8% myeloblasts and negative FC-MRD < 0.1%.

Following sustained hematological remission, a HAM (cytarabine 1000 mg/m^2^ every 12 h, days 1–3 + mitoxantrone 10 mg/m^2^/day, days 3 and 4) chemotherapy course was introduced. Unfortunately, around one month later control BM biopsy revealed 8.4% myeloblasts. A molecular analysis confirmed the presence of the *KMT2A::MLLT1* fusion gene and disclosed a positive FC-MRD. Considering the results, a disease relapse was diagnosed.

### 6.4. Case Approach and Results

The patient received first reinduction therapy, i.e., FLA-GO (fludarabine 30 mg/m^2^/day, days 1–5 + cytarabine 2000 mg/m^2^, days 1–5 + GO 4.5 mg/m^2^, day 4 and 7). Because of the bad treatment tolerance, such as profound BM aplasia leading to sepsis, the therapy was discontinued. Due to the achievement of hematologic remission, the decision was made to forgo the second cycle of FLA-GO treatment and proceed with hematopoietic stem cell transplantation. The patient underwent MUD peripheral blood stem cell transplant (PBSCT) CD34—7 × 10^6^/bw kg, CD3—3.5 × 10^8^/bw kg, preceded by myeloablative conditioning (fludarabine 30 mg/m^2^, days -7, -6, -5, -4, -3 + treosulfan 10 mg/m^2^, days -6, -5, -4 + thiotepa 2 × 5 mg/bw kg/day on day -2). Despite initially satisfactory graft success, follow-up bone marrow biopsies performed four months later showed FC-MRD with 16.73% myeloblasts and later 67.9% myeloblasts, revealing a post-transplant relapse of AML.

### 6.5. Discussion

Cases of coexisting similar genetic aberrations have been described among adults [196,197] and children [198] with AML. A complex karyotype (CK) in AML is defined as having three or more unrelated chromosomal abnormalities [21] in the absence of the WHO recurrent genetic aberrations of t(8;21)(q22;q22), inv(16)(p13.1q22)/t(16;16) (p13.1;q22); t(15;17)(q22;q12); t(9;11)(p22;q23), t(6;9)(p23;q34), inv(3)(q21q26)/t(3;3)(q21;q26), and t(9;22)(q34;q11.2) [199,200,201]. The prevalence of CK-AML varies from 9.5% to 18.5% [202,203]. Moreover, prevalence of CK in the pediatric AML population is age-dependent, with a higher incidence in children below the age of two (20.0%) than in young children and adolescents (10.8%) [202], which is similar to data regarding our patient. Although more research is needed to establish the role of CK in pediatric AML, recent studies show that patients with CK-AML have reduced EFS and OS rates compared with patients with intermediate-risk AML, indicating that CK is an unfavorable risk marker for pediatric AML [202]. However, for patients with a CK-AML and coexisting *KMT2A* rearrangement, the prognosis seems to be determined by the fusion partner gene rather than the karyotype structure [198].

## 7. Conclusions

In recent years, our comprehension of the pathophysiology of pediatric AML has expanded significantly. We now know that specific individual and disease-specific factors have predictive value. Although AML is a heterogeneous disease, understanding the underlying genetic background has led to the identification of recurrent genetic abnormalities, which form the basis of current patient risk stratification. Studies of clonal blast evolution are also underway to better understand the nature of disease relapse. All this has led to the development of new approaches, an improved ability to measure small numbers of malignant myeloid cells, and the introduction of targeted therapy with numerous newly approved drugs. Although various genetic lesions are already part of risk stratification in children with AML, many mutations are yet to be revealed. As we enter the era of genome-wide NGS to help us go beyond what is known today about clinically relevant genetic variations, with further implications for classification, prognosis, and therapy, collaboration on data and clinical trials is essential.

## Figures and Tables

**Table 1:** Summary of selected recently described germline mutations associated with the development of pediatric AML.

Gene	Region	Syndrome	Mean Age at Onset (Range and Average Age in Years)	Associated Malignancy/ies	References
*CEBPA*	19q13.1	Familial AML with mutated *CEBPA*	Early childhood to early adulthood (2 to 50, 25)	AML	[52,53,54,55,56,57]
*GATA2*	3q21.3	Familial MDS/AML with mutated *GATA2*/*GATA2* deficiency syndrome	Early teens to early twenties (<1 to 78, 20)	MDS/AML, CMML	[52,58,59,60,61]
*ETV6*	12p13.2	Thrombocytopenia 5	Childhood to early adulthood (2 to 82, 22)	MDS/AML, CMML, B-cell ALL, plasma cell neoplasm	[52,62,63,64,65,66,67,68]
*RUNX1*	21q22.12	Familial platelet disorder with propensity to myeloid malignancies	Early childhood to late adulthood (6 to 76, 33)	MDS/AML, T-cell ALL	[52,58,69,70,71,72,73,74]
*ANKRD26*	10p12.1	Thrombocytopenia 2	Childhood to early adulthood (1 to 84, 38)	MDS/AML, CML, MPN, ALL, CLL, MM	[75,76,77,78]
*TP53*	17p13.1	Li–Fraumeni syndrome	Childhood to late adulthood (20–70, 20)	MDS/AML, ALL, t-MN, lymphoma, MM, osteosarcoma, breast cancer, brain tumors, soft tissue sarcoma, adrenocortical carcinoma, and other solid tumors	[79,80,81,82,83]
*SAMD9/9L*	7q21.2	MIRAGE syndrome (*SAMD9*)*;* ataxia-pancytopenia syndrome (ATXPC) (*SAMD9L*)	Early childhood (0 to 2, 2)	MDS/AML, CMML	[52,84,85,86,87]

AML—acute myeloid leukemia, MDS—myelodysplastic syndrome, CMML—chronic myelomonocytic leukemia, ALL—acute lymphoblastic leukemia, t-MN—therapy-related myeloid neoplasm, MM—multiple myeloma, CLL—chronic lymphoblastic leukemia, MPN—myeloproliferative neoplasm, CML—chronic myeloid leukemia.

**Table 2:** Summary of selected studies on technologies used to assess MRD in AML.

Method	Sensitivity	Markers	Reference
Multiparameter Flow Cytometry (MPFC)	10^−3^ to 10^−5^	early progenitor markers: CD34, CD117 myeloid markers: CD11b, CD13, CD15, CD33 various differentiation markers: CD2, CD7, CD19, CD56, CD45, HLA-DR	[115,116,117,118,119]
Quantitative Polymerase Chain Reaction (qPCR)	10^−3^ to 10^−5^	*NPM1*, *RUNX1-RUNX1T1*, *CBFB-MYH11*, *PML-RARA*, *KMT2A-MLLT3*, *DEK-NUP214*, *BCR-ABL*, *WT1*	[120,121]
Next-Generation Sequencing (NGS)	10^−2^	*CALR*, *CEBPA*, *DDX41*, *ETV6*, *EZH2*, *FLT3*, *IDH1*, *IDH2*, *JAK2*, *KIT*, *KRAS*, *MPL*, *NPM1*, *NRAS*, *PTPN11*, *RAD21*, *RUNX1*, *SF3B1*, *SRSF2*, *STAG2*, *TP53*, *U2AF1*, *WT1*	[116,121]

**Table 3:** Risk stratification based on molecular and cytogenetic abnormalities occurring in pediatric AML.

Risk	Molecular Abnormalities	Cytogenetic Abnormalities	References
Favorable	t(8;21)(q22;q22.1)	*RUNX1::RUNX1T1*	[21]
	t(16;21)(q24;q22)	*RUNX1*::*CBFA2T3*	[144]
	inv(16)(p13.1q22) t(16;16)(p13.1;q22)	*CBFB*::*MYH11*	[125]
	t(9;11)(p22;q23)	*KMT2A*::*AF9 (MLLT3)*	[144]
	t(11;19)(q23;p13.1)	*KMT2A*::*ELL*	[144]
		*CEBPA* gene single or double mutations	[132]
		*NPM1*	[135]
	del(7q)		[144]
Intermediate	t(11;19)(q23;p13.3)	*KMT2A*::*MLLT1*	[140]
	t(10;11)(p12;q14)	*PICALM*::*MLLT10*	[144]
	t(8;16)(p11;p13)	*KAT6A*::*CREBBP*	[144]
	t(3;5)(q25;q35)	*NPM1*::*MLF1*	[144]
	t(1;22)(p13;q13)	*RBM15*::*MKL1*	[144]
		*FLT3* *-ITD*	[134]
Adverse	t(9;22)(q34;q11)	*BCR*::*ABL1*	[21]
	t(6;9)(p22;q34)	*DEK*::*NUP214*	[21]
	t(7;12)(q36;p13)	*ETV6::MNX1*	[144]
	inv(3)(q21.3q26.2) t(3;3)(q21.3;q26.2)	*GATA2*, *MECOM (EVI1)*	[144]
	t(9;11)(p21;q23)	*KMT2A*::*MLLT3*	[145]
	t(5;11)(q35;p15)	*NUP98*::*NSD1*	[144]
	t(11;12)(p15;p13)	*NUP98*::*KMD5A*	[144]
	t(16;21)(p11;q23)	*FUS::ERG*	[146]
	t(6;11)(q27;q23) t(4;11)(q21;q23) t(10;11)(p12;q23)	*KMT2A::AF6 (MLLT4)*	[147]

## Data Availability

The original contributions presented in this study are included in the article. Further inquiries can be directed to the corresponding author(s).

## Abbreviations

The following abbreviations are used in this manuscript:

AML	Acute Myeloid Leukemia
ALL	Acute Lymphoblastic Leukemia
NGS	Next-Generation Sequencing
HRG	High-Risk Group
MUD	Matched Unrelated Donor
SCT	Stem Cell Transplantation
OS	Overall Survival
NIH	National Institutes of Health
HSCT	Hematopoietic Stem Cell Transplantation
WBC	White Blood Cell
CNS	Central Nervous System
CR	Complete Remission
FAB	French–American–British
PFS	Progression-Free Survival
BM	Bone Marrow
EFS	Event-Free Survival
BMI	Body Mass Index
RFS	Relapse-Free Survival
DIC	Disseminated Intravascular Coagulation
TLS	Tumor Lysis Syndrome
CBF	Core-Binding Factor
WES	Whole-Exome Sequencing
MRD	Measurable Residual Disease
DFS	Disease-Free Survival
MPFC	Multiparameter Flow Cytometry
qPCR	Quantitative Polymerase Chain Reaction
ddPCR	Digital Droplet Polymerase Chain Reaction
ELN	European Leukemia Net
GO	Gemtuzumab Ozogamicin
SMMHC	Smooth Muscle Myosin Heavy Chain
NPM1	Nucleophosmin 1
*FLT3*-ITD	*FLT3*-Internal Tandem Duplications
*FLT3*-TKD	*FLT3*-Tyrosine Kinase Domain
allo-HSCT	allogenic HSCT
TRM	Treatment-Related Mortality
FDA	Food and Drug Administration
COG	Children’s Oncology Group
HAR	High Allelic Ratio
CR1	First Complete Remission
GVL	Graft Versus Leukemia
GVHD	Graft-Versus-Host Disease
TBI	Total Body Irradiation
BuCy	busulfan–cyclophosphamide
BuCyMel	busulfan–cyclophosphamide–melphalan
CloFluBu	clofarabine–fludarabine–busulfan
TDM	Therapeutic Drug Monitoring
LFS	Leukemia-Free Survival
MAC	Myeloablative Conditioning
RIC	Reduced-Intensity Conditioning
DLI	Donor Lymphocyte Infusion
BCL-2	B-Cell Leukemia/Lymphoma-2
JM	Juxtamembrane
MCL-1	Myeloid Cell Leukemia 1
BH3	BCL-2 homology 3
MOMP	Mitochondrial Outer Membrane Permeabilization
HMA	Hypomethylating Agent
HDAC	Histone Deacetylase
HDACi	Histone Deacetylases inhibitor
AZA	Azacitidine
DEC	Decytabine
HATs	Histone Acetyltransferases
MHC	Major Histocompatibility Complex
NK	Natural Killer
CAR-T	Chimeric Antigen Receptor T cell
ADCs	Antibody–Drug Conjugates
BiTE	Bispecific T-cell Engager
DART	Dual-Affinity Retargeting
MNs	Modal Numbers
ctDNA	Circulating Tumor DNA
cfDNA	Circulating Free Cell DNA
miRNA-29a	microRNA-29a
miRNA-100	microRNA-100
CRGs	Cuproptosis-Related Genes
FC-MRD	Flow-Cytometry-Measurable Residual Disease
WHO	World Health Organization
ISCN	International System of Human Cytogenomic Nomenclature
AIEOP-BMF	Associazione Italiana Ematologia Oncologia Pediatrica-Berlin, Frankfurt, Münster
PBSCT	Peripheral Blood Stem Cell Transplantation
CK	Complex Karyotype
